# Zebrafish *polg2* knock-out recapitulates human POLG-disorders; implications for drug treatment

**DOI:** 10.1038/s41419-024-06622-9

**Published:** 2024-04-20

**Authors:** Raquel Brañas Casas, Alessandro Zuppardo, Giovanni Risato, Alberto Dinarello, Rudy Celeghin, Camilla Fontana, Eleonora Grelloni, Alexandru Ionut Gilea, Carlo Viscomi, Andrea Rasola, Luisa Dalla Valle, Tiziana Lodi, Enrico Baruffini, Nicola Facchinello, Francesco Argenton, Natascia Tiso

**Affiliations:** 1 Department of Biology, University of Padova, Padova, 35131 Italy; 2 Department of Biomedical Sciences, University of Padova, Padova, 35131 Italy; 3 Department of Cardio-Thoraco-Vascular Sciences and Public Health, University of Padova, Padova, 35128 Italy; 4 Novo Nordisk Foundation Center for Stem Cell Medicine (reNEW), University of Copenhagen, Copenhagen, 2200 Denmark; 5 Department of Animal and Aquatic Sciences, Faculty of Agriculture, Chiang Mai University, Chiang Mai, 50200 Thailand; 6 Department of Chemistry, Life Sciences and Environmental Sustainability, University of Parma, Parma, 43124 Italy; 7 Neuroscience Institute, Italian Research Council (CNR), 35131 Padova, Italy

**Keywords:** Disease model, Neuromuscular disease

## Abstract

The human mitochondrial DNA polymerase gamma is a holoenzyme, involved in mitochondrial DNA (mtDNA) replication and maintenance, composed of a catalytic subunit (POLG) and a dimeric accessory subunit (POLG2) conferring processivity. Mutations in *POLG* or *POLG2* cause POLG-related diseases in humans, leading to a subset of Mendelian-inherited mitochondrial disorders characterized by mtDNA depletion (MDD) or accumulation of multiple deletions, presenting multi-organ defects and often leading to premature death at a young age. Considering the paucity of POLG2 models, we have generated a stable zebrafish *polg2* mutant line (*polg2*^*ia304*^) by CRISPR/Cas9 technology, carrying a 10-nucleotide deletion with frameshift mutation and premature stop codon. Zebrafish *polg2* homozygous mutants present slower development and decreased viability compared to wild type siblings, dying before the juvenile stage. Mutants display a set of *POLG*-related phenotypes comparable to the symptoms of human patients affected by POLG-related diseases, including remarkable MDD, altered mitochondrial network and dynamics, and reduced mitochondrial respiration. Histological analyses detected morphological alterations in high-energy demanding tissues, along with a significant disorganization of skeletal muscle fibres. Consistent with the last finding, locomotor assays highlighted a decreased larval motility. Of note, treatment with the Clofilium tosylate drug, previously shown to be effective in POLG models, could partially rescue MDD in Polg2 mutant animals. Altogether, our results point at zebrafish as an effective model to study the etiopathology of human POLG-related disorders linked to POLG2, and a suitable platform to screen the efficacy of POLG-directed drugs in POLG2-associated forms.

## Introduction

Mitochondrial DNA (mtDNA) depletion syndromes (MDS) are a large group of hereditary diseases characterized by autosomal recessive or dominant transmission, both clinically and genetically heterogeneous [1]. The common features underlying MDS consist of impaired cell bioenergetics, altered mtDNA maintenance and replication, and a severe mtDNA depletion (MDD), which results in dysfunction of high ATP consuming organs [2, 3]. Indeed, the most impacted tissues in MDS comprise brain, skeletal muscle, liver, kidney and intestine. MDS can be caused by mutations in nuclear genes involved either in nucleotide biosynthesis (*TK2*, *SUCLA2*, *SUCLG1*, *RRM2B*, *DGUOK*, and *TYMP*), in nucleotide transport (*MPV17* and *SLC25A4/ANT1*) or mtDNA replication (*POLG*, *POLG2*, *SSBP1*, *TWNK*, *TFAM*, *LIG3* and *MGME1*) [4]. More recently, mitochondrial fusion was also involved in mtDNA replisome maintenance, since altered fusion processes, caused in most cases by mutations in *OPA1*, also lead to MDD [5]. In addition, mutations in other genes have been reported in few cases in MDS, such as *AGK*, *FBXL4*, *MRM2*, *SLC25A21*, *SLC25A10/DIC* [4].

Among the autosomal mitochondrial disorders, those related to mtDNA polymerase gamma (Pol-γ or Pol gamma) dysfunction are the most common and best studied. The Pol gamma is the only replicative polymerase so far known to function in animal mitochondria [6], being essential for mtDNA replication and repair. The holoenzyme of human Pol gamma consists of a catalytic subunit (p140) encoded by *POLG* (at chromosomal locus 15q25) and a homodimeric form of its accessory subunit (p55), encoded by *POLG2* (at chromosomal locus 17q24.1) [7]. The POLG catalytic subunit is a 140 kDa protein with 5′-3′ DNA polymerase, 3′-5′ exonuclease and 5′ dRP lyase (5′-deoxyribose-5-phosphate lyase) activities, while the accessory subunit POLG2 is a 55 kDa protein acting as a homodimer for tight DNA binding and processive DNA synthesis. Studies on HeLa cells demonstrated that the POLG2 subunit increases by 50 to 100 times the incorporation of nucleotides if compared with the isolated catalytic subunit, enhancing the Pol gamma DNA processivity [8]. Moreover, it has recently been published that POLG2 is also able to directly bind to DNA, providing new insights into molecular defects associated with POLG2 disruption in mitochondrial disease [9].

Mutations in human *POLG* or *POLG2* lead to a group of multi-organ mitochondrial diseases with Mendelian inheritance, collectively named POLG-related disorders [10], characterized by multiple deletions and/or depletion of mtDNA. *POLG* mutations have been linked to a continuum of overlapping phenotypes including Alpers-Huttenlocher syndrome, childhood myocerebrohepatopathy spectrum, myoclonic epilepsy myopathy sensory ataxia, ataxia neuropathy spectrum, cardiomyopathy, autosomal recessive and autosomal dominant progressive external ophthalmoplegia (arPEO and adPEO) [10]. To date, most patients bearing *POLG2* mutations have been diagnosed with adPEO, variably displaying additional manifestations including skeletal muscle weakness, ataxia, depression and various neuro-sensory symptoms, typically worsening with the passing of the years. Current POLG-related disorder treatments are non-curative and symptom-focused. Interestingly, Clofilium tosylate (CLO) has shown efficacy in ameliorating POLG-related symptoms in yeast, worm, and cell-based models [11], as well as in zebrafish mutants [12]. CLO is classified as an anti-arrhythmic agent of Class III due to its potassium (K^+^) channel-blocking properties [11]. CLO is a quaternary ammonium compound, and the active site of the molecule is clofilium, which, being positively charged, can insert itself into the potassium channel, thereby blocking its exit from the cell. This interference impedes repolarisation in phase III of the cardiac action potential, leading to an increased refractory period and providing an anti-arrhythmic effect. While this mechanism is well-established, its applicability under *polg2* mutations remains unexplored.

Several model organisms have been employed to study Pol-γ malfunctioning, including *S. cerevisiae*, *D. melanogaster*, *C. elegans*, and *M. musculus*. Mouse Pol-γ is highly similar to the human holoenzyme, representing a very good system for studying human *POLG/POLG2* pathogenic variants and POLG-related disorders. However, knock-out (KO) of *POLG* in mice causes nearly complete loss of mtDNA and lethality before reaching an adult stage [13]. Therefore, despite their genetic similarity to humans, murine models present some limitations as model organisms since *Polg*^*-/-*^ mutants lose viability during embryogenesis [14]. Conversely, recent studies conducted on *D. rerio* point at zebrafish as a valuable model to study human *POLG/POLG2* pathogenic variants, given that mutations in zebrafish *polg* resulted in viable larval individuals, with significant mtDNA depletion, slower development and decreased mitochondrial mass, similarly to what observed in human patients [12, 15]. The zebrafish Polg protein is highly conserved with the human POLG enzyme, with a 70% level of identity and 79% of similarity. Furthermore, zebrafish Polg2 shows a 50% identity to the human orthologue, with a 67% level of similarity. Furthermore, the zebrafish holoenzyme stoichiometry reflects the human polymerase gamma conformation, with a single Polg catalytic polypeptide interacting with two Polg2 accessory subunits. Unlike mouse models, zebrafish *polg*^*−/−*^ mutant lines have been shown to phenocopy many traits observed in human POLG-related disorders [12, 15], such as MDD, growth delay and premature death. These features make zebrafish a suitable model organism to study clinical characteristics of POLG-related diseases, and to evaluate potential therapeutic compounds able to rescue the phenotypic traits of the mutants.

Considering the current paucity of Polg2 models, this study aims at better understanding the consequences of Polg2 dysfunction, seeking to reveal some phenotypic traits of disease progression in vivo. Specifically, we have focused on the morpho-functional characterization of a novel zebrafish *polg2* knock-out (KO) line, *polg2*^*ia304*^, with a 10-nucleotide deletion in exon 4, leading to a premature stop codon. This line represents the first zebrafish *polg2* mutant, with potential application in POLG disease modelling.

## Material and methods

### *Danio rerio* husbandry

All experiments were performed in accordance with the Italian and European Legislations (Directive 2010/63/EU) [13] and with permission for animal experimentation from the Ethics Committee of the University of Padua and the Italian Ministry of Health (Authorization number D2784.N.DQT). Zebrafish (1:1 sex ratio) were reared under standard conditions, following the procedures recommended in the Zebrafish Book [16]; growth parameters were set as described by Spence and colleagues [17]. The environment was kept at 28.5 °C controlled temperature in a 12:12 light–dark (LD) cycle. Fish were fed following international guidelines [18, 19].

For anaesthesia or euthanasia of zebrafish embryos and larvae, tricaine (MS222; E10521, Sigma-Aldrich) was added to the fish water at 0.16 mg/mL or 0.3 mg/mL, respectively. Wild-type (wt) lines used in this work included Tuebingen, Giotto and Umbria strains. For mitochondria live imaging, the mitochondria-expressed *Tg(Hsa.Cox8a:MLS-EGFP)*^*ia301*^ transgene was used [12, 20]. For in vivo assessment of hypoxia signalling, the Hif-Hypoxia signalling reporter *Tg(4xHRE- TATA:EGFP)*^*ia21*^ was used [21]. Liver was analysed under confocal microscopy using the *Tg(lfabf:dsRed;elaA:EGFP)*^*gz15*^ [22] line.

For all experiments, heterozygous animals were crossed and their progenies grouped, blinded analysed and, later, genotyped.

### Generation and genotyping of the zebrafish *polg2* KO mutant line

The zebrafish mutant line *polg2*^*ia304*^ was generated by CRISPR/Cas9-mediated genome editing. A single guide RNA (sgRNA) was designed using the CHOPCHOP software (https://chopchop.rc.fas.harvard.edu) to specifically target an optimal CRISPR sequence on exon 4 of *polg2* gene (XM_001921095). The chosen sgRNA (Supplementary Table 1) was synthesized following Gagnon et al. [23] and transcribed in vitro using the MEGAshortscript T7 kit (AM1354, Life Technologies). One-cell stage embryos were injected with 2 nL of a solution containing 280 ng/μL of Cas9 protein (M0646, New England Biolabs) and 68 ng/μL of sgRNA; 0.05% phenol red was used as an injection marker. F0 injected embryos were raised to adulthood and screened, by F1 genotyping, for germline transmission of the mutation. Heterozygous mutants, harbouring the mutation of choice, were outcrossed 4 times to remove undesired mutations, and then incrossed to obtain homozygous mutants (F5 generation). Screening primers for heterozygous and homozygous fish (Supplementary Table 1) were designed with Primer3 software (https://primer3.ut.ee) to amplify a 137-bp region across the *polg2* sgRNA target region. The *polg2*^*ia304*^ mutant line bears a 10-nucleotide deletion in exon 4 of *polg2* gene (allele ia304), with reading frameshift that leads to a premature stop codon. In mutants, the amino acid (aa) sequence of Polg2 is altered after the aa 235, and the mutated protein displays a 267-aa length. PCR products were separated in ethidium bromide-stained 3.5% low EEO agarose gel (BP160-500, Fisher BioReagents) to identify *polg2*^*+/+*^, *polg2*^*+/ia304*^ and *polg2*^*ia304/ia304*^ genotypes.

### Survival analysis

Pools of wild type (wt), heterozygous and homozygous larvae were obtained from a *polg2*^*+/ia304*^ incross and raised in different tanks under the same conditions (water, light, food). Alive zebrafish in each tank were counted every 5 days; the resulting proportions were compared with the expected Mendelian ratios.

### Histological analysis

Zebrafish larvae were euthanized at 20 dpf (days post fertilization) and placed in 24-well plates (one larva per well). Each individual was placed in 500 μL of Bouin fixative solution for 6 h. Larvae were rinsed in 70% ethanol (EtOH) to remove picric acid and later stored at 4 °C in this solution until the dehydration step. Once larvae were genotyped, samples were dehydrated in increasing concentrations of ethanol, infiltrated with xylene and embedded in Paraplast plus (39602004, Leica). Sectioning was performed using a Rotary One (LKB) microtome to obtain 8-μm-thick slices. After rehydration, the sections were stained with haematoxylin and eosin and mounted with Eukitt (09-00100, Bio Optica) for microscopic analysis.

### Transmission electron microscopy (TEM) analysis

Larvae at 20 dpf were fixed with 2.5% glutaraldehyde (16220, EMS) plus 2% paraformaldehyde (P6148, Sigma-Aldrich) in 0.1 M sodium cacodylate buffer pH 7.4 O/N at 4 °C. Subsequently, the samples were postfixed with 1% osmium tetroxide (19130, EMS) in 0.1 M sodium cacodylate buffer for 1 h at 4 °C. After three water washes, samples were dehydrated in a graded ethanol series and embedded in an epoxy resin (46345, Sigma-Aldrich). Ultrathin sections (60-70 nm) were obtained with a Leica Ultracut EM UC7 ultramicrotome, counterstained with uranyl acetate and lead citrate and viewed with a Tecnai G2 (FEI) transmission electron microscope operating at 100 kV. Images were captured with a Veleta (Olympus Soft Imaging System) digital camera.

### Developmental analysis

Body length of individuals was measured for all genotypes (*polg2*^*+/+*^, *polg2*^*+/ia304*^ and *polg2*^*ia304/ia304*^) at different developmental stages: 3, 6 and 20 dpf. Larvae were mounted in 2% methylcellulose (in fish water) and bright-field imaged under a Leica M165FC microscope equipped with a DFC7000T digital camera (Leica). Body length measurements, taken sagittally from the tip of the head to the caudal fin upper end, were performed on the acquired images using the ImageJ software. Adults at 3 months post-fertilization (mpf) were measured on grid paper upon Tricaine anesthetization.

### Mitochondrial DNA analysis

*polg2*^*+/+*^, *polg2*^*+/ia304*^ and *polg2*^*ia304/ia304*^ larvae were collected at 6 dpf and 20 dpf to analyse mtDNA relative quantity by real-time qPCR. While a small section of the tail was cut to perform the genotyping, the fish body was stored in lysis buffer (100 mM Tris HCl pH 8-8.5, 200 mM NaCl, 0.2% SDS, 5 mM EDTA) and proteinase K (10 mg/mL, V3021, Promega), at -20 °C. After the genotyping, individuals were grouped together by genotype, creating pools of 10 fish. Each pool was kept overnight at 55 °C to release the DNA from the cell lysate. Total DNA was extracted by the phenol-chloroform method: 2.5 volumes of 100% EtOH and 1/10 volume of 3 M sodium acetate (NaAc) were used to precipitate DNA. Precipitated DNA was later washed with 500 μL of 70% EtOH, left to dry and re-suspended in 15 μL of nuclease-free water. DNA quantification was performed using the NanoDrop system (NanoDrop 2000, Thermo Scientific). Samples were stored at -20 °C until analysis.

### Gene expression analysis

Total RNA was extracted from pools of 10 larvae (*polg2*^*+/+*^, *polg2*^*+/ia304*^ and *polg2*^*ia304/ia304*^) at 6 dpf using TRIzol Reagent (15596018, Invitrogen), quantified by NanoDrop system (NanoDrop 2000, Thermo Scientific) and reverse-transcribed using the High-Capacity cDNA Reverse Transcription Kit (Applied Biosystems, Thermo Fisher Scientific). Primer sequences are listed in Supplementary Table 1. All primers were designed using the software Primer3.

### Behavioural assay

Motility assay was performed at 6, 15, and 20 dpf, using the DanioVision system (Noldus Information Technology) and the EthoVision XT software. Larvae were placed in 24-well plates with 500 μL of fish water per well and allowed to rest for 20 min under light conditions at 28 °C. After an additional 20 min of acclimation at 28 °C in a dark environment inside the DanioVision chamber, larvae were exposed to three alternating periods of white light (100% illumination, 5000 lux) and dark (0% illumination, <1 lux), as previously described in MacPhail et al. [24]. A more detailed protocol is shown in Supplementary Fig. 1 (A-B).

Tapping analysis was performed at 8 dpf, following a protocol adapted from that described in Faria et al. [25]. Larvae were placed in 24-well plates with 500 μL of fish water per well and allowed to rest for 20 min under light conditions at 28 °C. After 5 minutes of acclimation at 28 °C in a light condition environment, larvae were exposed to a tapping stimulus every second at maximum intensity level in a period of white light (100% illumination, 5000 lux. A more detailed protocol is shown in Supplementary Fig. 1A, C.

### Heart rate analysis

To evaluate cardiac function under *polg2* mutation, heart rate was assessed in vivo at 2, 3, 4, and 8 dpf. Larvae were positioned in 24-well plates containing 500 μL of fish water per well, anaesthetized with 0.16 mg/mL Tricaine and left 10 min at 28 °C for acclimation. Heart beats were counted three times per individual within 3 independent replicates and expressed as beats per minute (bpm).

### Mitochondria assessment in vivo

To assess mitochondrial mass and shape, *polg2*^*+/ia304*^ adults, bearing the mitoGFP *Tg(Hsa.Cox8a:MLSEGFP)*^*ia301*^ transgene, were inter-crossed. The fluorescent offspring was mounted in 1% agar (in fish water) and imaged using a Nikon Eclipse 90i microscope in a C2 confocal system. Signal quantification was performed using ImageJ software and results were normalized twice, using area and average integrated density, in comparison with wt controls, as described in Facchinello et al. [26].

### Mitochondrial membrane potential analysis

Mitochondrial membrane potential (Δψ) was investigated in zebrafish embryos by means of the fluorescent probe tetramethylrhodamine methyl ester (TMRM). Zebrafish embryos at 3 dpf were incubated for 24 h in fish water medium with 300 nM TMRM and 1.6 μM CsH (cyclosporin H, needed to inhibit the multidrug resistance pump), as previously described by Stocco et al. [27]. At 4 dpf, fish were mounted in depression slides in 1% low-melting agarose (in fish water) and subjected to confocal microscope scanning.

### Mitochondrial metabolism analysis

The Agilent Seahorse Extracellular Flux Analyzer was used to measure oxygen consumption rate (OCR). Embryos at 4 dpf were placed in a 24-well plate (one individual per well), leaving four wells empty to control possible temperature fluctuations across the plate. On the top of the Seahorse plate, mitochondrial drugs (FCCP, antimycin A, and rotenone) were pre-injected to be progressively released during the ongoing analysis. First measurements allowed to assess the baseline OCR and derive the basal respiration levels. Then, oligomycin was added (complex V inhibitor) to measure ATP-linked respiration and proton leak. FCCP protonophore was instead employed to collapse the inner membrane gradient and allow the electron transport chain (ETC) to function at its maximal rate (maximum respiration). Finally, antimycin A and rotenone (complex III and I inhibitors, respectively) were added to block the ETC, revealing the non-mitochondrial respiration. Altered parameters and lower rates of respiration were associated with metabolic dysfunction and pathological states.

### Drug treatment with clofilium tosylate

Clofilium tosylate (CLO) drug treatment was performed on embryos from *polg2*^*ia304/+*^ incross. Embryos were exposed to 3 μM CLO, during 5 days (from 2 dpf to 6 dpf). The toxicity of CLO was previously evaluated and found to be non-toxic below a concentration of 5 μM [12]. The efficiency of the CLO drug treatment was evaluated as the ability to rescue decreased mtDNA content. The mtDNA was quantified by qPCR analysis, as described in Facchinello et al. [12]. In parallel, additional embryos from *polg2*^*ia304/+*^ incross were raised in the facility at standard conditions [14] until they reached 10 dpf, when they were treated with 2 μM CLO, during 6 days (from 10 to 15 dpf). The efficiency of the CLO drug treatment at larval stage was evaluated as the ability to rescue the motility phenotype detected in *polg2*^*ia304*^ mutants, using the DanioVision system (Noldus Information Technology) and the EthoVision XT software, as detailed in the *Behavioural assay* section.

### Statistical analysis

All statistical analyses were performed using GraphPad Prism (GraphPad Software Inc.). The sample size was preliminarily calculated by G*Power and Sample Size Calculator analysis. Sample final sizes were obtained after collection and randomization from multiple mating events, excluding unfertilized eggs or embryos displaying very early developmental defects in all conditions. All data are derived from three independent single-blind experiments, each with independent biological replicates. The Shapiro–Wilk normality test was employed to assess the normal distribution of the data (α = 0.05). For the data that passed the normality test, unpaired t-tests or ordinary one-way ANOVA were applied. Multiple comparisons were corrected using Tukey’s test after ordinary one-way or two-way ANOVA. In cases where data did not pass the normality test, non-parametric tests such as Mann-Whitney or Kruskal-Wallis tests were utilized. The latter was employed for multiple comparisons, followed by Dunn’s test. Barlett’s test was employed to examine the homoscedasticity of samples. If samples passed this test (p > 0.05), ordinary ANOVA analyses were conducted. In contrast, when data showed heteroscedasticity, the Games-Howell’s multiple comparisons test was employed. Contingency analysis was performed using the *χ*^2^ test. Results are expressed as the mean ± SEM, and statistical significance was considered when *p* < 0.05 (*), *p* < 0.005 (**), *p* < 0.001 (***) and *p* < 0.0001 (****). The statistical tests used for single experiments are mentioned in the corresponding figure legends. The exact value of sample size (n) is reported in figure legends. For gene expression and relative mtDNA quantification assays, pools of 10 larvae were considered if ≤6 dpf, whereas single larvae were analysed for ≥20 dpf. For in vivo experiments, the sample size represents the number of animals analysed.

## Results

### *polg2* KO zebrafish survive until larval stage

**Fig. 1:**
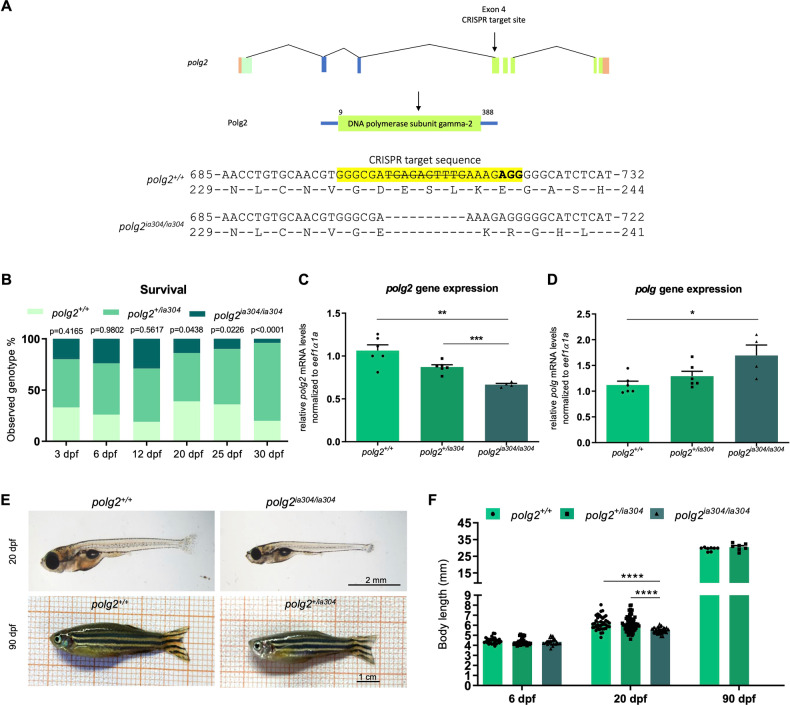
*polg2*^*ia304*^ line generation, survival and developmental delay. **A** Schematic representation of zebrafish *polg2* and its editing by CRISPR/Cas9. **B** *polg2*^*ia304/ia304*^ individuals fail to survive to 4 weeks post fertilization. Data were analysed by χ2 test; *p* = 0.4165; *p* = 0.9802; *p* = 0.5617; *p* = 0.0438; *p* = 0.0226; *p* < 0.0001; 3 dpf (n = 120), 6 dpf (n = 115), 12 dpf (n = 89), 20 dpf (n = 70), 25 dpf (n = 50), 30 dpf (n = 46). Gene expression analysis performed to assess *polg2* (**C**) and *polg* (**D**) mRNA levels in *polg2*^*ia304*^ mutant line. Values are reported as mean ± SEM of three independent experiments and analysed by Games-Howell’s multiple comparison test (**C**) and ordinary one-way ANOVA followed by Tukey’s test (**D**); *p < 0.05; **p < 0.005; ***p < 0.001; *polg2*^*+/+*^ (n = 6 × 10-larva pools), *polg2*^*+/ia304*^ (n = 6 × 10-larva pools), *polg2*^*ia304/ia304*^ (n = 4 × 10-larva pools). **E** Representative images of zebrafish *polg2*^*+/+*^ control and *polg2*^*ia304/ia304*^ mutant at 20 dpf, and *polg2*^*+/+*^ control and a *polg2*^*+/ia304*^ heterozygote at 90 dpf. Scale bar: 2 mm (top); scale bar: 1 cm (bottom). **F** Body length analysis in wt (*polg2*^*+/+*^), heterozygous (*polg2*^*+/ia304*^) and homozygous (*polg2*^*ia304/ia304*^) individuals. Values are expressed as mean ± SEM. Statistics were assessed by Mann-Whitney test; ****p < 0.0001; *polg2*^*+/+*^ 6 dpf (n = 25), *polg2*^*+/ia304*^ 6 dpf (n = 41), *polg2*^*ia304/ia304*^ 6 dpf (n = 22); *polg2*^*+/+*^ 20 dpf (n = 39), *polg2*^*+/ia304*^ 20 dpf (n = 74), *polg2*^*ia304/ia304*^ 20 dpf (n = 45); *polg2*^*+/+*^ 90 dpf (n = 8), *polg2*^*+/ia304*^ 90 dpf (n = 7).

By applying the CRISPR/Cas9 technology, a zebrafish *polg2* mutant line was generated, named *polg2*^*ia304*^. The line bears a 10-nucleotide deletion in exon 4, leading to a premature stop codon (Fig. 1A and Supplementary Fig 2A–D). This KO mutation was continuously outcrossed over five generations, to dilute potential non-specific lesions in other genomic regions. Survival analysis showed that the *polg2*^*ia304*^ line is larval vital but juvenile lethal under homozygous condition. From 5 dpf to 15 dpf the observed distribution of the genotypes did not differ significantly from the expected 25:50:25 Mendelian ratios. However, at 20 dpf the number of *polg2* homozygous mutant larvae was significantly lower than expected. Only a single *polg2*^*ia304/ia304*^ individual could survive up to 30 dpf, the early juvenile stage (Fig. 1B). As expected, the expression of *polg2* was significantly decreased in *polg2*^*ia304/ia304*^ mutants compared to *polg2*^*+/+*^ siblings, probably due to the nonsense mediated RNA decay process (Fig. 1C). Conversely, the expression of the paralogous gene *polg* was increased in *polg2*^*ia304/ia304*^ mutants (*p* < 0.05) compared to *polg2*^*+/+*^ controls, suggesting the occurrence of genetic compensation, a phenomenon usually observed in zebrafish knockouts [28] (Fig. 1D).

### *polg2* KO affects zebrafish growth

To further characterize macroscopic effects of the *polg2*^*ia304*^ mutation, differences in body development were investigated, observing that 20 dpf homozygous *polg2*^*ia304*^ mutants displayed a significantly reduced body length when compared to wt and heterozygous siblings. Wild type and heterozygous individuals did not show significant differences in development, either at 6, 20 and 90 dpf (Fig. 1E, F).

### *polg2* KO impairs locomotor activity

**Fig. 2:**
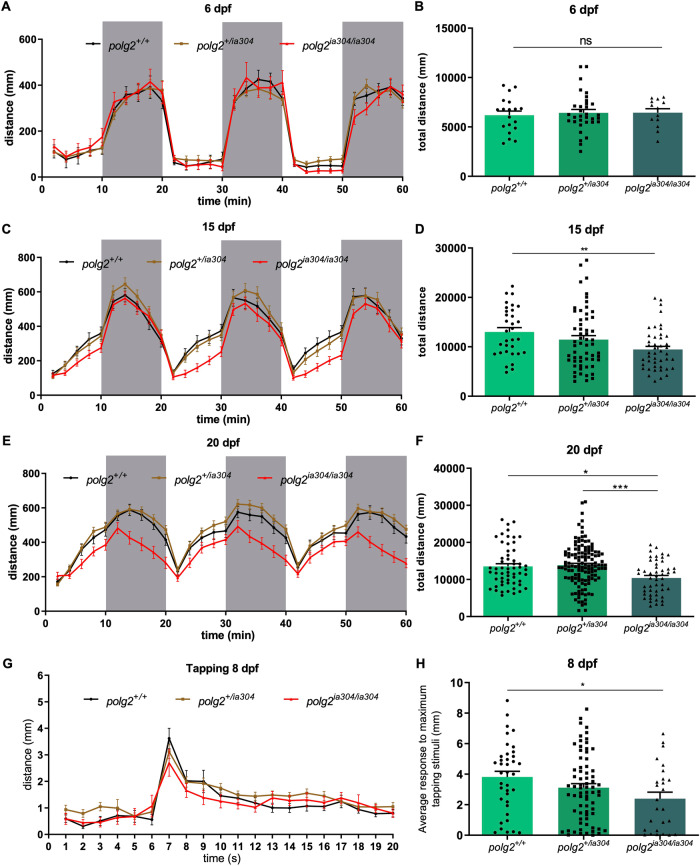
*polg2*^*ia304*^ behavioural defects assessed by locomotor analysis. **A**, **C**, **E** Effect of light stimuli on locomotion in *polg2*^*ia304/ia304*^ mutants at 6, 15 and 20 dpf, respectively. White fields indicate light exposition time, while grey fields represent dark periods. The analysis assessed the average total distance swum at 6 dpf (**B**), 15 dpf (**D**) and 20 dpf (**F**). Values derived from 3 independent biological replicates and are reported as mean ± SEM. Data were analysed using ordinary one-way ANOVA and Tukey’s test (**B**) or Kruskal-Wallis together with Dunn’s multiple comparison test (**D**, **F**); *p < 0.05; **p < 0.005; ***p < 0.001; for 6 dpf *polg2*^*+/+*^ (n = 19), *polg2*^*+/ia304*^ (n = 33), *polg2*^*ia304/ia304*^ (n = 13); for 15 dpf *polg2*^*+/+*^ (n = 32), *polg2*^*+/ia304*^ (n = 58), *polg2*^*ia304/ia304*^ (n = 45); for 20 dpf *polg2*^*+/+*^ (n = 57), *polg2*^*+/ia304*^ (n = 131), *polg2*^*ia304/ia304*^ (n = 49). **G** Effect of tapping stimulus on locomotion in wt, heterozygous and *polg2*^*ia304/ia304*^ individuals at 8 dpf. Tapping stimuli were delivered in a 20-s time window with an interstimulus interval of 1 s. **H** Average response to maximum tapping stimuli given as the total distance covered by *polg2*^*ia304/ia304*^ homozygotes during tap stress assay, compared to wt and heterozygous sibs. Results are expressed as mean ± SEM. For statistical analysis, the Kruskal-Wallis test corrected with Dunn’s multiple comparison test was applied; *p < 0.05; *polg2*^*+/+*^ (n = 38), *polg2*^*+/ia304*^ (n = 72), *polg2*^*ia304/ia304*^ (n = 26), from 3 independent experiments.

Being the skeletal muscle one of the most affected tissues in POLG patients, we investigated the locomotor behaviour of 6, 15 and 20 dpf zebrafish larvae when exposed to alternating light and dark conditions. When testing 6 dpf larvae, locomotor patterns evoked by light-dark and dark-light transitions almost overlap for the three genotypes and reflect the pattern expected for zebrafish larvae (Fig. 2A). The total distance covered did not significantly differ among the three genotypes at 6 dpf (Fig. 2B). When performing the motility assay on 15- and 20-dpf larvae (Figs. 2C and 2E, respectively), the light/dark response is maintained for the three genotypes and corresponds to the previously described pattern. However, there is a statistically significant decrease in the total distance covered by *polg2*^*ia304/ia304*^ mutants compared to wt controls at both 15 (Fig. 2D) and 20 dpf (Fig. 2F), suggesting preserved sensory abilities but progressively impaired motor response in homozygous mutants. In parallel, analysis under tapping stimuli confirmed preserved sensibility but altered motility (Fig. 2G), being the average response to maximum tapping stimuli significantly lower in *polg2*^*ia304*^ mutants when compared to wt siblings (Fig. 2H), indicating that the peripheral sensory system is working normally but the motorial one is impaired. We thus decided to investigate if metabolic and/or structural alterations could underlie these locomotor phenotypes.

### *polg2* KO leads to histological alterations in high energy-demanding tissues

To investigate how *polg2*^*ia304*^ mutation ultimately impacts on tissue organization of homozygous mutants, we performed histological analysis on zebrafish larvae at advanced developmental stage (20 dpf).

Based on previous evidence (ZFIN database), *polg2* is known to be ubiquitously expressed in wt adult tissues. However, we focused the histological analysis on high energy demanding organs: brain, heart, skeletal muscle, liver and gut, which represent tissues frequently affected in POLG-related disorders, and where mitochondria contribution to the cell energy supply is more prominent.

**Fig. 3:**
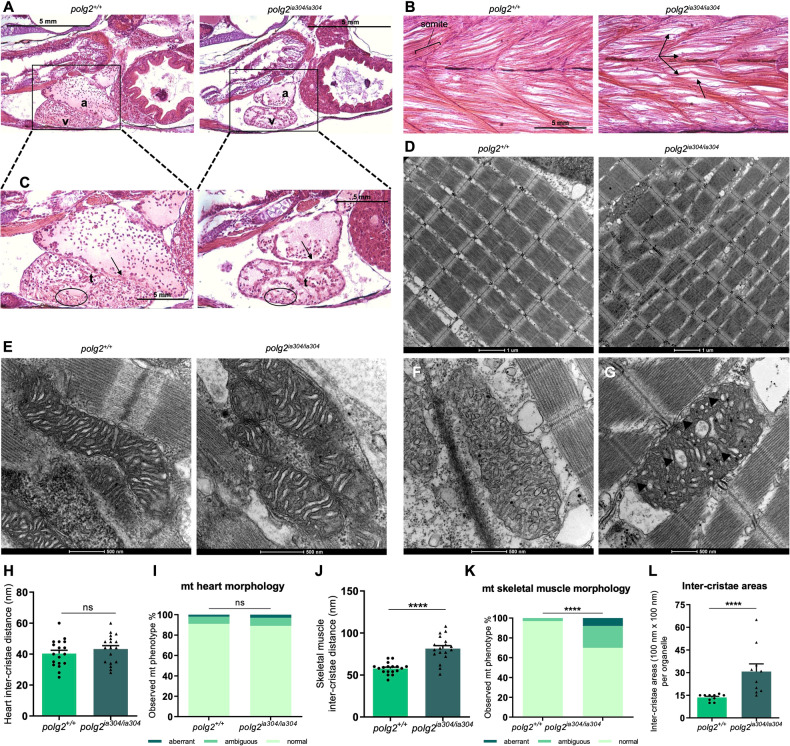
Effects of *polg2* mutation on high energy demanding tissues. **A**, **C** Comparison of cardiac tissues at 20 dpf, reporting differences in atrial (a) and ventricular (v) dimensions and an altered trabecular network (t) in mutants. Scale bar: 5 mm. **B** Histological sections of *polg2*^*+/+*^ and *polg2*^*ia304/ia304*^ skeletal muscle tissue at 20 dpf, displaying disorganized fibres and altered somite boundaries in mutants. Arrows: detachment of skeletal muscle fibres. Scale bar: 5 mm. **D** TEM analysis of 20 dpf skeletal muscle of wt (*polg2*^+/+^) and homozygous mutants (*polg2*^*ia304/ia304*^), the latter displaying altered organization of myofibrils. Scale bar: 1 µm. **E** TEM analysis of mitochondria in 20 dpf heart of wt (*polg2*^+/+^) and homozygous mutants (*polg2*^*ia304/ia304*^). Scale bar: 500 nm. TEM analysis of 20 dpf skeletal muscle from wt (*polg2*^+/+^) (**F**) and homozygous mutants (*polg2*^*ia304/ia304*^) (**G**) confirms altered morphology of myofibrils and mitochondrial cristae (black arrowheads) in mutants. Scale bar: 500 nm. **H** Quantification of inter-cristae distance (in nm) in the cardiac region. Data are shown as mean ± SEM and statistics were done using unpaired Student’s t-test. **I** Quantification of heart mitochondrial (mt) aberrant morphologies. For statistical analysis, the χ^2^ test was applied; N = 8 independent TEM images per condition, in triplicate. **J** Quantification of inter-cristae distance (in nm) in the skeletal muscle. Values are reported as mean ± SEM. Statistics were assessed using unpaired Student’s t-test; ****p < 0.0001. **K** Quantification of skeletal muscle mt aberrant morphologies. χ^2^ test was used for statistical analysis, N = 8 independent TEM images per condition, in triplicate. ****p < 0.0001. **L** Number of wide inter-cristae areas, considered when at least equal to 100 × 100 nm, counted for each organelle in the skeletal muscle. Data are shown as mean ± SEM. Mann-Whitney test was used for statistics; ****p < 0.0001.

The mutant heart displayed a thinner and more rarefied trabecular network compared to wt controls (Fig. 3A, C). Moreover, the homozygous mutant heart had reduced dimensions compared to the wt, albeit maintaining a normal heart rate until 8 dpf (Supplementary Fig. 3A). Concerning the skeletal muscle, *polg2*^*ia304/ia304*^ muscle fibres appeared ragged and less organized compared to wt controls (Fig. 3B). This disorganization was already detectable at 3 dpf by birefringence assay (Supplementary Fig. 3B, C) and worsened with later stages of development.

Ultrastructural observations were made by transmission electron microscopy (TEM) on heart and skeletal muscle of 20-dpf larvae. No significant differences were detectable when analyzing mitochondria of the cardiac region (Fig. 3E). Mutant heart mitochondria appeared similar to wt ones, with regular inter-cristae distance (Fig. 3H) and mitochondrial morphology (Fig. 3I). However, TEM analyses in the skeletal muscle revealed that *polg2*^*ia304/ia304*^ mutants present an altered organization of myofibrils, which lose their characteristic symmetrical arrangement and appear partially disrupted, compared to wt (Fig. 3D), confirming what observed by histological analysis. Regarding the mitochondrial morphology in the skeletal muscle (Fig. 3F, G), TEM analyses revealed a significant increase of mitochondria with aberrant cristae architecture in *polg2*^*ia304/ia304*^ mutant muscles (Fig. 3J). Specifically, unlike wt mitochondria, where tubular-like cristae are homogeneously distanced, *polg2*^*ia304/ia304*^ mitochondria presented wider inter-cristae areas (Fig. 3K), in larger amount compared to normal organelles (Fig. 3L).

We also inspected the histological phenotype of liver and gut of 20 dpf *polg2*^*ia304/ia304*^ zebrafish (Supplementary Fig. 4A), but no pathological abnormalities could be detected in comparison with histological samples of wt siblings. We did not either detect a sign of liver failure at 6 dpf (Supplementary Fig. 4B, C), whereas *polg* ablation leads to significant defects in this organ [12]. As far as concerns the encephalic region, histological sections of the brain (Supplementary Figure 5A) revealed a significantly reduced organ size in mutants. All size measurements were evaluated after normalization to the body length (Supplementary Fig. 5C), being measured from histological images inter-comparable in terms of sagittal section (Supplementary Fig. 5B).

### *polg2* KOs display altered mitochondrial network

**Fig. 4:**
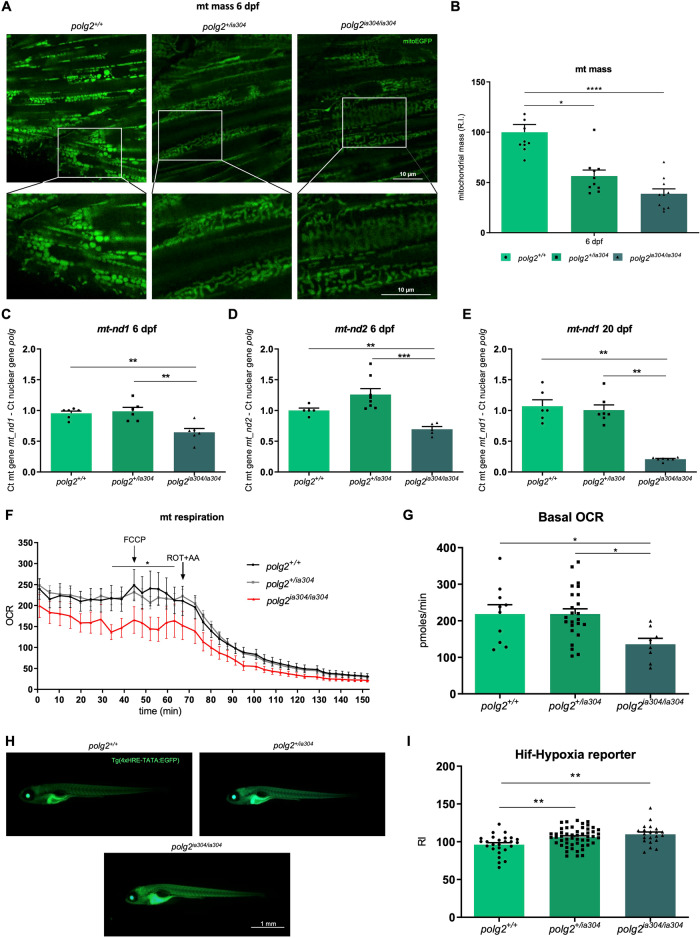
Defects in mitochondrial content, morphology and metabolism under *polg2* KO. **A** Confocal images of the *Tg(Hsa.Cox8a:MLS-EGFP)*^*ia301*^ mitochondrial marker (*mito:EGFP*) in *polg2*^*+/+*^, *polg2*^*+/ia304*^ and *polg2*^*ia304/ia304*^ zebrafish at 6 dpf. Scale bar: 10 µm. **B** Relative quantification of the *mito:EGFP* transgene at 6 dpf. Data are reported as mean ± SEM. Statistics were assessed using the Kruskal-Wallis test followed by Dunn’s multiple comparison test; *p < 0.05; ****p < 0.0001; n = 10 independent larvae per condition. Comparison of relative abundances of the mitochondrial *nd1* (**C**) and *nd2* (**D**) genes in wt (*polg2*^*+/+*^), heterozygous (*polg2*^*+/ia304*^) and homozygous (*polg2*^*ia304/ia304*^) zebrafish at 6 dpf. Values are shown as mean ± SEM. Ordinary one-way ANOVA (**C**) or Games-Howell’s multiple comparison test (**D**) were applied for statistics; **p < 0.005; ***p < 0.001; **C** *polg2*^*+/+*^ (n = 6 × 10-larva pools), *polg2*^*+/ia304*^ (n = 6 × 10-larva pools), *polg2*^*ia304/ia304*^ (n = 6 × 10-larva pools); (**D**) *polg2*^*+/+*^ (n = 5 × 10-larva pools), *polg2*^*+/ia304*^ (n = 8 × 10-larva pools), *polg2*^*ia304/ia304*^ (n = 5 × 10-larva pools) from 3 independent biological replicates. **E** Analysis of the *nd1* gene at 20 dpf. Values are reported as mean ± SEM. For statistical analysis, the Kruskal-Wallis test corrected with Dunn’s multiple comparison test was used; **p < 0.005; *polg2*^*+/+*^ (n = 6), *polg2*^*+/ia304*^ (n = 7), *polg2*^*ia304/ia304*^ (n = 7) from 3 independent experiments. **F** Diagram depicting the oxygen consumption rate (OCR) profile by Seahorse assay in 4 dpf wt (*polg2*^*+/+*^), heterozygous (*polg2*^*+/ia304*^) and homozygous (*polg2*^*ia304/ia304*^) larvae under basal conditions, FCCP (carbonyl cyanide-p-trifluoromethoxyphenylhydrazone)-induced maximal respiratory capacity stimulation and ROT/AA (Rotenone/Antimycin A)-mediated inhibition. Two-way ANOVA followed by Tukey’s multiple comparison test was applied for statistical significance; *p < 0.05 between *polg2*^*+/+*^ and *polg2*^*ia304/ia304*^ individuals. **G** Quantification of basal OCR in 4 dpf zebrafish. Data are shown as mean ± SEM and were analysed by ordinary one-way ANOVA; *p < 0.05; *polg2*^*+/+*^ (n = 9), *polg2*^*+/ia304*^ (n = 24), *polg2*^*ia304/ia304*^ (n = 8) from 3 independent replicates. **H** Imaging of *HRE:EGFP* (Hif-hypoxia) reporter at 6 dpf: an example of wt (above, left), heterozygous (above, right) and homozygous (below) mutant larva. Scale bar: 1 mm. **I** Integrated density analysis of Hif-hypoxia signalling activation in 6-dpf wt (*polg2*^*+/+*^), heterozygous (*polg2*^*+/ia304*^) and homozygous (*polg2*^*ia304/ia304*^) larvae. Values are expressed as mean ± SEM and analysed by ordinary one-way ANOVA; **p < 0.005; *polg2*^*+/+*^ (n = 6), *polg2*^*+/ia304*^ (n = 26), *polg2*^*+/ia304*^ (n = 52), *polg2*^*ia304/ia304*^ (n = 21), coming from 3 independent experiments.

A significant mitochondrial mass decrease, evaluated with a *mito:EGFP* transgene at the skeletal muscle level (Fig. 4A), was found in *polg2*^*ia304/ia304*^ mutants and, interestingly, also in *polg2*^*+/ia304*^ heterozygotes, suggesting that *polg2* mutation in one allele is sufficient to affect the mitochondrial mass. At 6 dpf, the detected fluorescence signal in wt fish was twice as strong compared to *polg2*^*+/ia304*^ (*p* < 0.05) and *polg2*^*ia304/ia304*^ (*p* < 0.0001) signals (Fig. 4B).

Moreover, imaging of skeletal muscle mitochondria in 6 dpf transgenic larvae revealed a defective mitochondrial network (Fig. 4A), suggesting altered mitochondrial dynamics in *polg2*^*ia304/ia304*^ mutants, with fusion and fission processes not properly occurring. Specifically, the fluorescence signal was weaker in *polg2*^*+/ia304*^ individuals and even blurrier in *polg2*^*ia304/ia304*^ compared to wt (Fig. 4B), quantified as reported in Facchinello et al. [12].

### *polg2* KO leads to severe mtDNA depletion

Considering the importance of Polg and Polg2 proteins in MDD, and given the previously described alterations in the mitochondrial network upon *polg2* genetic ablation, we investigated the mtDNA content in the *polg2*^*ia304*^ line to evaluate whether MDD was present in mutants. At 6 dpf, a significant MDD was detected in *polg2*^*ia304/ia304*^ homozygotes (*p* < 0.005) while heterozygous and wt individuals maintained normal mtDNA content (Fig. 4C-D). The scenario was even worse at 20 dpf, with *polg2*^*ia304/ia304*^ mutants presenting severe MDD (*p* < 0.005) (Fig. 4E).

### Impaired mitochondrial bioenergetics under *polg2* KO

We further analysed if the severe MDD, detected in the *polg2*^*ia304*^ mutant line, could result in altered mitochondrial metabolism. To this purpose, the oxygen consumption rate (OCR) was measured using an Agilent Seahorse XF Extracellular Flux Analyzer. During the whole experiment, *polg2*^*+/ia304*^ heterozygotes behaved in a similar way compared to wt larvae, whereas *polg2*^*ia304/ia304*^ homozygotes displayed a distinct performance (Fig. 4F). Specifically, basal OCR in *polg2*^*ia304/ia304*^ individuals was lower compared to *polg2*^*+/ia304*^ and *polg2*^*+/+*^ siblings (Fig. 4G), and this difference in OCR was maintained also upon FCCP stimulation. Moreover, the OCRs among analysed genotypes tended to coincide after rotenone and antimycin A (ROT + AA) administration, suggesting that non-mitochondrial respiration is almost the same in all genotypes, while basal and maximal mitochondrial respiration rates are severely reduced in *polg2*^*ia304/ia304*^ mutants (*p* < 0.05).

Dysfunctional mitochondria can activate mt-to-nucleus retrograde signalling pathways, including hypoxia sensing, and changes in these signals are reported to correlate with mtDNA levels [29]. We therefore investigated whether *polg2*^*ia304*^ mutants could activate the Hif-mediated hypoxia signalling, taking advantage of the zebrafish *Tg(4xHRE-TATA:EGFP)*^*ia21*^ reporter line (Fig. 4H). Interestingly, 6-dpf *polg2*^*ia304/ia304*^ mutants exhibited significantly higher fluorescence intensities compared to wt (Fig. 4I), similarly to what previously observed in the *polg*^*ia302*^ zebrafish mutant line. Moreover, this activation could be observed also under heterozygous condition, suggesting than one mutant *polg2* allele is enough to trigger Hif-mediated responses.

A TMRM assay was also performed, to assess the status of the mitochondrial membrane potential in the *polg2*^*ia304*^ mutant line. The analysis did not reveal any significant difference among genotypes, although a decreased signal trend was observed in the mutants (Supplementary Fig. 6A, B).

### CLO can partially rescue mtDNA content in *polg2* KO mutants

**Fig. 5:**
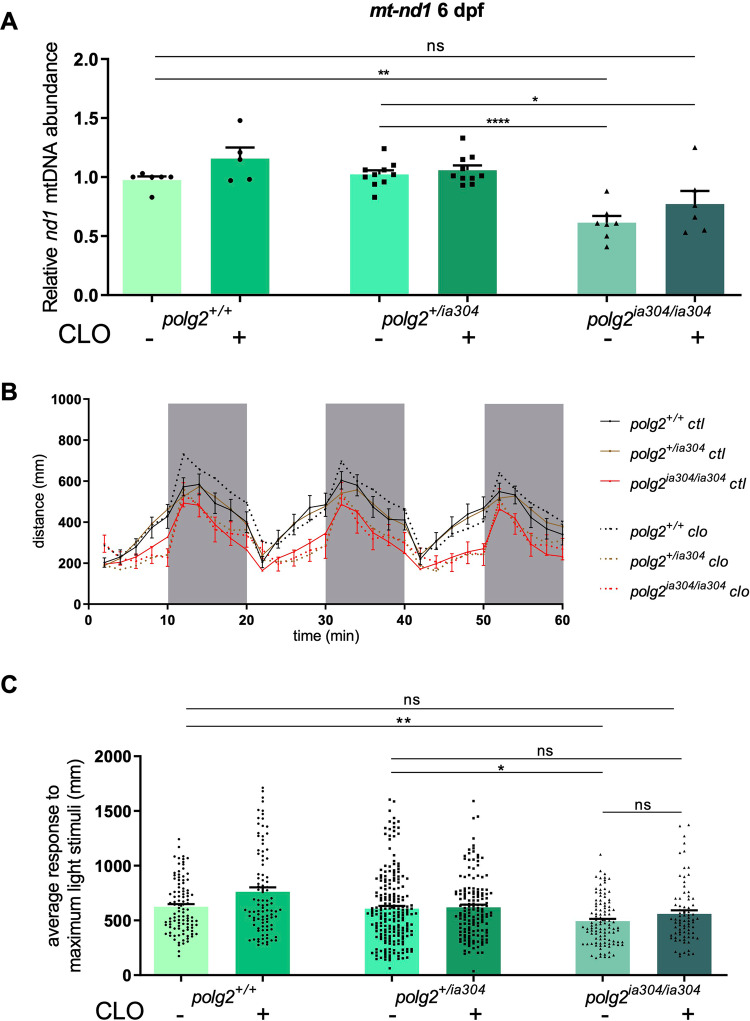
Analysis of CLO rescuing effects under *polg2* KO. **A** mtDNA content in treated (+) and non-treated (-) *polg2*^*+/+*^, *polg2*^*+/ia304*^ and *polg2*^*ia304/ia304*^ individuals, exposed to 3 μM CLO from 2 dpf to 6 dpf; Data are shown as mean ± SEM, each dot corresponds to a 10-fish pool. Statistics were assessed using ordinary one-way ANOVA, followed by Tukey’s multiple comparison test; *p < 0.05; **p < 0.005; ****p < 0.0001; *polg2*^*+/+*^ (n = 6 ×10-larva pools), *polg2*^*+/+*^ + CLO (n = 5 x 10-larva pools), *polg2*^*+/ia304*^ (n = 10 x 10-larva pools), *polg2*^*+/ia304*^ + CLO (n = 10 x 10-larva pools), *polg2*^*ia304/ia304*^ (n = 7 x 10-larva pools), *polg2*^*ia304/ia304*^ + CLO (n = 6 ×10-larva pools), from 3 independent biological replicates. **B** Effect of light stimuli on locomotion in treated (+) and non-treated (-) *polg2*^*+/+*^, *polg2*^*+/ia304*^ and *polg2*^*ia304/ia304*^ individuals, exposed to 2 μM CLO from 10 dpf to 15 dpf. White fields indicate light exposition time, while grey fields represent dark periods. Two-way ANOVA followed by Dunn’s multiple comparison test was applied for statistical significance; *polg2*^*+/+*^ (n = 34), *polg2*^*+/+*^ + CLO (n = 32), *polg2*^*+/ia304*^ (n = 70), *polg2*^*+/ia304*^ + CLO (n = 50), *polg2*^*ia304/ia304*^ (n = 35), *polg2*^*ia304/ia304*^ + CLO (n = 26). **C** Maximum response to light stimuli, expressed in mm (distance swum) among the three genotypes (*polg2*^*+/+*^, *polg2*^*+/ia304*^ and *polg2*^*ia304/ia304*^) in treated (+) and non-treated (-) fish. Values are reported as mean ± SEM. Statistical analysis was done by Kruskal-Wallis test followed by Dunn’s multiple comparison test; **p < 0.005; *p < 0.05; *polg2*^*+/+*^ (n = 34), *polg2*^*+/+*^ + CLO (n = 32), *polg2*^*+/ia304*^ (n = 70), *polg2*^*+/ia304*^ + CLO (n = 50), *polg2*^*ia304/ia304*^ (n = 35), *polg2*^*ia304/ia304*^ + CLO (n = 26), from 3 independent replicates and in triplicate.

The recent demonstration of rescuing effects using the Clofilium tosylate (CLO) drug in Polg-deficient zebrafish [12] prompted us to test if this molecule could be effective also under Polg2 mutant condition. Similar to what observed for *polg* mutants [11], we detected a positive effect of CLO on mtDNA content. Specifically, while mtDNA content was significantly lower in *polg2* KO compared to wt, this difference disappeared upon CLO treatment, suggesting that CLO, although not having the strong effect previously observed in *polg*^*sa9574*^ [12], could partially recover the MDD in the absence of Polg2 (Fig. 5A).

We assessed the impact of CLO treatment on locomotor behaviour in 15-dpf zebrafish larvae. Notably, CLO treatment demonstrated a slight enhancement in the swimming performance of *polg2*^*ia304/ia304*^ homozygotes (Fig. 5B). Despite this improvement, the swimming performance did not fully reach normal levels, likely attributed to the severity of the *polg2* KO condition. Conversely, CLO treatment showed no discernible effect on heterozygous mutants, potentially due to inherent variability in their responses. Intriguingly, homozygous mutants exhibited an improvement in responsiveness under light stimuli when treated with CLO, achieving comparable results to the wt control siblings (Fig. 5C).

## Discussion

The paucity of experimental systems concerning Polg2 dysfunction has until now hindered modelling and pharmacological treatment of POLG-related phenotypes linked to Polg2.

We describe here the generation and morpho-functional characterization of a zebrafish *polg2* KO line; the *polg2*^*ia304*^ allele bears a 10-nucleotide deletion leading to a frameshift mutation and premature stop codon. The lack of antibodies targeting zebrafish Polg2 did not allow protein quantifications in the mutants. However, the decrease of *polg2* mRNA and the increase of *polg* expression suggest mechanisms of mutation-induced mRNA decay for *polg2*, and compensatory over-expression of the *polg* member.

The lethality of *polg2* homozygous mutants between 3–4 weeks post fertilization (wpf), before the juvenile phase, demonstrates the essential role for Polg2 in organism survival, further corroborating the hypothesis of a loss-of-function effect for the induced mutation. We tend to exclude dominant negative or critical haploinsufficiency effects for the *polg2*^*ia304*^ allele, since heterozygous individuals are viable and fertile, although with a decreased mitochondrial mass compared to wt controls.

*polg2* homozygotes vitality during larval stages allowed the identification of a series of features resembling phenotypes observed in human patients with early onset POLG-related disorders [3, 30] including reduced survival, developmental delay and histological changes in energy-demanding organs, progressive locomotor defects, MDD, altered mitochondrial architecture and reduced OCR, all attributable to a dysfunctional mtDNA replisome machinery.

Decreased mtDNA gene copies available for ETC subunit synthesis are likely to generate a fall in membrane potential of the organelle and, therefore, a reduction in ATP synthesis [31]. Furthermore, this mutation leads to decreased mitochondrial mass, modified mitochondrial network and impaired mitochondrial function. In fact, mitochondrial metabolism was assessed by Seahorse assay in *polg2*^*ia304/ia304*^ mutants, detecting reduced OCR, in agreement with the presence of mitochondrial bioenergetic defects. Moreover, impaired mitochondrial function and MDD already emerged at 6 dpf in *polg2*^*ia304/ia304*^ mutants, while reduced growth and altered behaviours were appreciable only later during development, suggesting that the slower growth was a consequence of the impaired bioenergetics and the lower amount of ATP available for cell processes. The impairment in bioenergetics may explain the altered development of high-energy demanding tissues in zebrafish and other organisms, including humans, underlying defects in brain, liver, muscles and heart, often associated with MDS and POLG-related disorders [10, 32]. On the other hand, Hif-hypoxia biosensor imaging suggested a robust activation of this pathway in mtDNA-depleted *polg2* mutants. The observed changes raise the question on which mechanism could elicit such mt-to-nucleus retrograde signalling. Indeed, besides low O_2_ levels, multiple cues of mitochondrial dysfunction can trigger upregulation of the Hif pathway, including a “pseudo-hypoxia” state [12] due to a compromised cellular ability to use O_2_ in spite of its availability.

In-depth analysis of a selected set of tissues evidenced histological changes that could underlie developmental delay and premature death of *polg2*^*ia304/ia304*^ individuals. For instance, *polg2* homozygous mutants are characterized by an improper skeletal muscle development, resulting in disorganized fibres and somite disruption, further leading to a defective locomotor behaviour. Of note, human mitochondrial diseases are characterized by altered skeletal muscle function which leads to the mitochondrial myopathies reported in MDS and POLG-related disorders [33].

At the cardiac level, histological analysis of *polg2*^*ia304/ia304*^ mutants detected decreased atrium and ventricle dimensions compared to wt, reflecting a reduced development of heart muscle and altered trabecular network, which might recall the cardiomyopathies often reported in mitochondrial disorders [34]. This feature is also congruent with a reduced development of other high-energy demanding tissues in *polg2* mutants, as oxygen supply is expected to be compromised by systemic cardiovascular insufficiency.

As for the neural system, measurements obtained from histological brain sections revealed a remarkably smaller size of mutant encephalon compared to wt, suggesting impaired neurological development in *polg2*^*ia304/ia304*^ individuals, as seen in Alpers-Huttenlocher syndrome and other POLG-related disorders [10, 35]. Together with the skeletal muscle defects, this aspect may contribute, at least in part, to the reduced locomotor response to light/dark transitions observed in *polg2* mutants under motility assay.

Taking advantage of previous studies identifying Clofilium tosylate (CLO) as a candidate drug for the treatment of POLG-related disorders [11, 12], we exposed *polg2*^*ia304*^ mutant individuals to the CLO molecule during early development, assessing the mtDNA relative content between treated and non-treated individuals. As predicted, CLO slightly increased the mtDNA basal levels in wt controls and, noteworthy, this effect was also observed in *polg2* heterozygous and homozygous mutants. Moreover, CLO appeared to improve the responsiveness of *polg2*^*ia304*^ mutants under light stimuli, revealing once again its potentiality as an effective drug for POLG-related disorders. Indeed, this evidence suggests that CLO treatment may represent a therapeutic strategy not only for Polg dysfunction but also in case of Polg2 mutation, widening the range of mitochondrial conditions potentially tractable by CLO.

Along the same lines, it would be interesting to test longer drug treatment periods in these zebrafish mutant lines and evaluate if CLO is able to rescue other phenotypic traits such as mitochondrial mass, mitochondrial metabolism, mt-to-nucleus retrograde signalling, development of high-energy demanding tissues, and locomotor activity. On the other hand, similar to what observed for *polg* mutants [12], we detected a positive effect of CLO on mtDNA content, raising the basal levels in both wt and *polg2*^*+/ia304*^ heterozygotes. Of note, CLO was able to increase mtDNA content also in *polg2*^*ia304/ia304*^ homozygous mutants, after five days of treatment (Fig. 5A). However, CLO effect appears limited under very severe conditions, when Polg or Polg2 subunits are lacking, when compared to KD/point mutant models [12]. This fact strengthens the hypothesis that the CLO mechanism of action is the stabilization of the DNA Polymerase gamma holoenzyme, organized as heterotrimeric complex in vertebrates.

In conclusion, the analysis of the first zebrafish *polg2* KO line revealed peculiar phenotypes recalling those observed also in Polg (Polg1) mutants such as severe mtDNA depletion, altered mitochondrial dynamics, reduced growth, impaired locomotor activity and premature death, all faithfully modelling features observed in human POLG patients. This study highlights the importance of the polymerase gamma accessory subunit (Polg2) in zebrafish mtDNA replication and organism survival, since homozygous *polg2*^*ia304*^ mutations drastically affect larval development and lifespan. Recent studies identified Polg2 as a key protein in the maintenance of mtDNA D-loop, displaying dsDNA binding activity other than solely acting as a processivity factor [9]. This double role of Polg2 in mtDNA replication and maintenance explains why mutations in *polg2* result in severe MDD and lethal phenotypes, as observed in *polg2*^*ia304/ia304*^ mutants. Thus, it would be interesting to further analyse this aspect in future studies, dissecting the role of Polg2 in D-loop formation and maintenance.

Finally, this study provides a proof-of-principle that drugs pre-identified in Polg-targeted screens, such as CLO, may be effective in rescuing Polg2-dependent phenotypes. This opens the way to the exploitation of zebrafish *polg2* mutants (this KO and future KI lines) as additional platforms for drug screening in search of a therapy for POLG-related disorders and other MDD diseases.

### Supplementary information

Supplementary-Material-clear

aj-checklist

## Data Availability

Fish lines used in this study are available through an MTA.
