# Supplementary material for: Zebrafish polg2 knock-out recapitulates human POLG-disorders; implications for drug treatment
Source: Cell Death Dis. 2024 Apr 20;15(4):281. doi: 10.1038/s41419-024-06622-9 (PMC11032366; doi:10.1038/s41419-024-06622-9)
Supplement: Supplementary file 1 — Supplementary-Material-clear [file 41419_2024_6622_MOESM1_ESM.pdf]

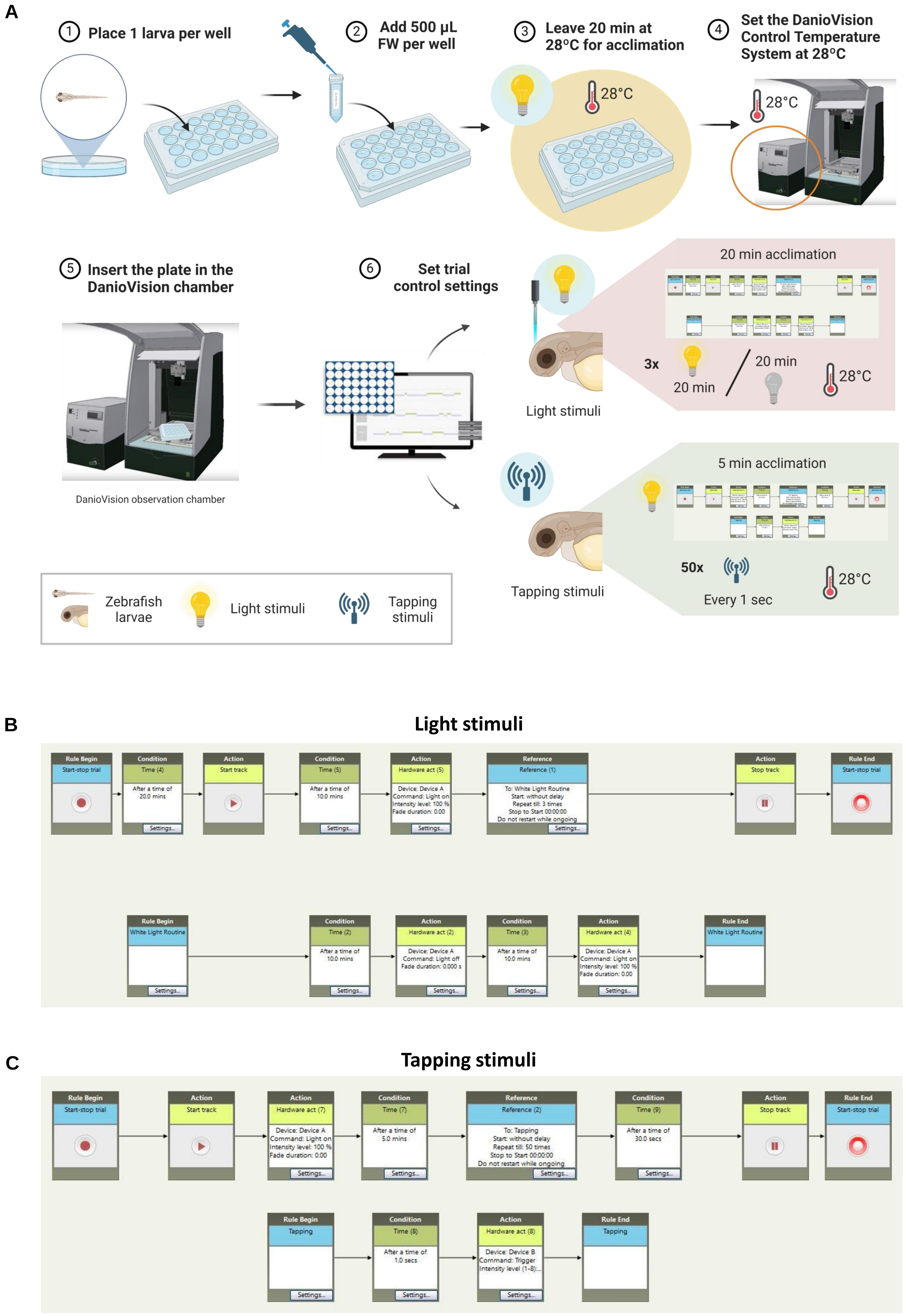
A
1 Place 1 larva per well
2 Add 500 µL FW per well
3 Leave 20 min at 28°C for acclimation
4 Set the DanioVision Control Temperature System at 28°C
28°C
28°C
5 Insert the plate in the DanioVision chamber
DanioVision observation chamber
6 Set trial control settings
Light stimuli
20 min acclimation
3x
20 min
20 min
28°C
5 min acclimation
Tapping stimuli
50x
Every 1 sec
28°C
Zebrafish larvae
Light stimuli
Tapping stimuli
B
Light stimuli
Rule Begin
Start-stop trial
Condition
Time (4)
After a time of 20.0 mins
Action
Start track
Condition
Time (5)
After a time of 10.0 mins
Action
Hardware act (5)
Device: Device A
Command: Light on
Intensity level: 100 %
Fade duration: 0.00
Reference
Reference (1)
To: White Light Routine
Start: without delay
Repeat till: 3 times
Stop to Start 00:00:00
Do not restart while ongoing
Action
Stop track
Rule End
Start-stop trial
Rule Begin
White Light Routine
Condition
Time (2)
After a time of 10.0 mins
Action
Hardware act (2)
Device: Device A
Command: Light off
Fade duration: 0.000 s
Condition
Time (3)
After a time of 10.0 mins
Action
Hardware act (4)
Device: Device A
Command: Light on
Intensity level: 100 %
Fade duration: 0.00
Rule End
White Light Routine
C
Tapping stimuli
Rule Begin
Start-stop trial
Action
Start track
Action
Hardware act (7)
Device: Device A
Command: Light on
Intensity level: 100 %
Fade duration: 0.00
Condition
Time (7)
After a time of 5.0 mins
Reference
Reference (2)
To: Tapping
Start: without delay
Repeat till: 50 times
Stop to Start 00:00:00
Do not restart while ongoing
Condition
Time (9)
After a time of 30.0 secs
Action
Stop track
Rule End
Start-stop trial
Rule Begin
Tapping
Condition
Time (8)
After a time of 1.0 secs
Action
Hardware act (8)
Device: Device B
Command: Trigger
Intensity level (1-8):
Rule End
Tapping

Suppl. Figure 1: DanioVision protocol setting for behavioural analysis in zebrafish larvae

(A) Flowchart protocol for behavioural analysis in zebrafish larvae using a 24-well plate. Temperature was set to 28°C with the DanioVision Temperature Control Unit. FW: Fish Water. Created with BioRender.com. (B) Trial Control Settings for the light stimuli protocol. (C) Trial Control Settings for the tapping stimuli protocol.

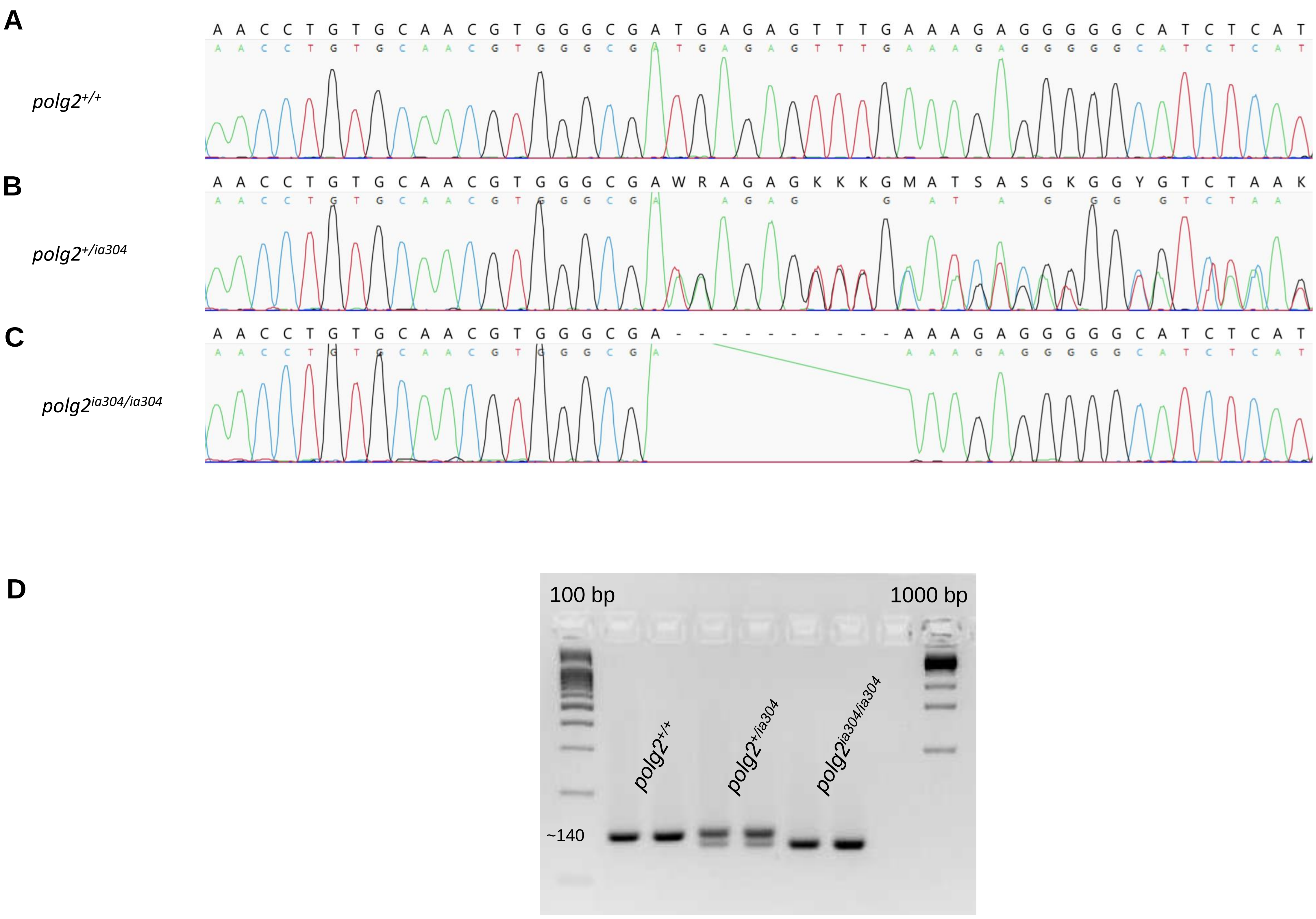

Suppl. Figure 2: Genotyping of the zebrafish *polg2^{ia304}* mutant line

(A-C) Chromatograms corresponding to *polg2$^{+/+}$*(A), *polg2$^{+/ia304}$* (B) and *polg2$^{ia304/ia304}$* (C) individuals, aligned with SeqMan Ultra, DNASTAR Lasergene. (D) Representative gel image of PCR genotyping using genomic DNA from tail fins of larvae from a cross between *polg2$^{+/ia304}$* heterozygotes.

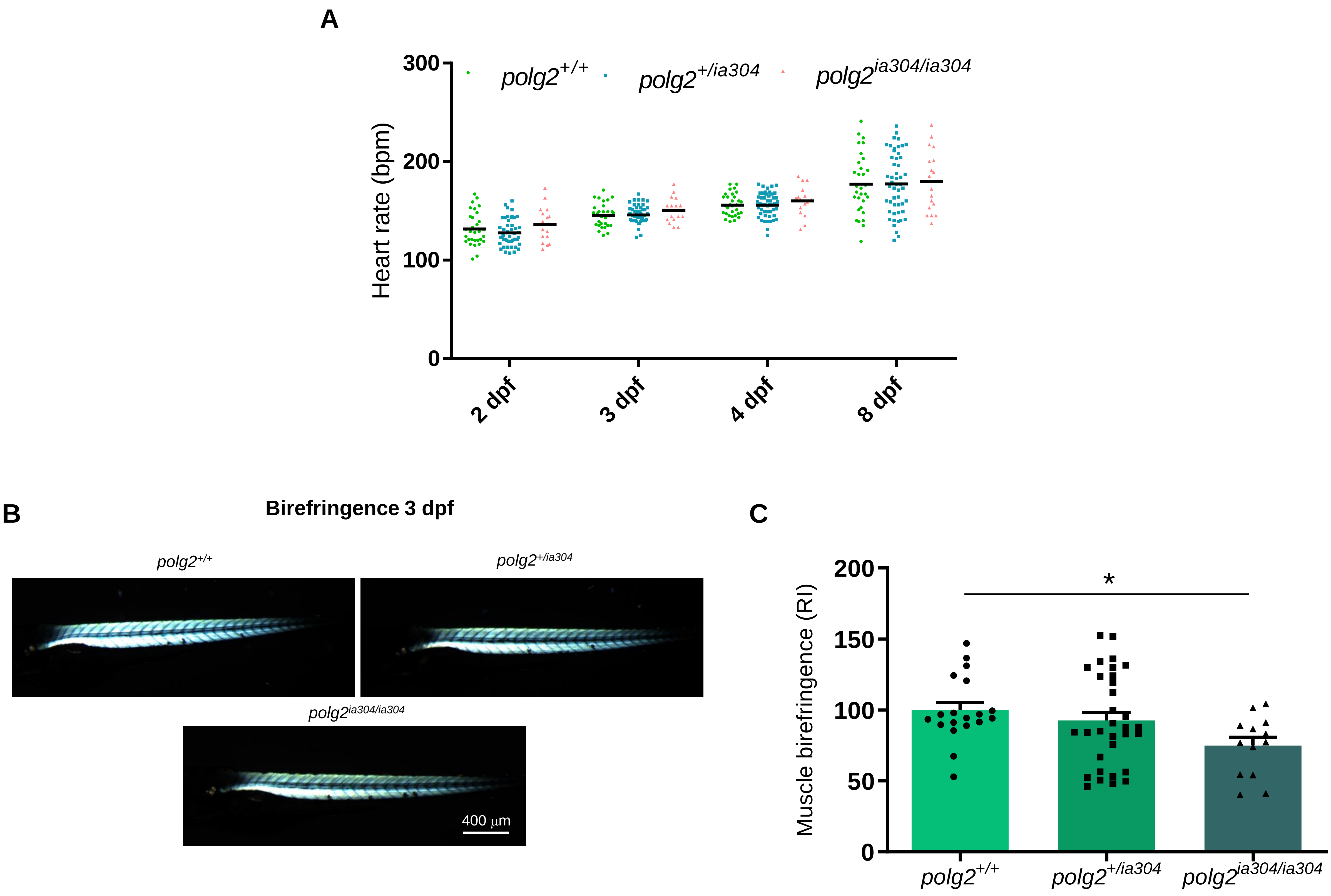

Suppl. Figure 3: Analysis of heart rate and skeletal muscle organization in *polg2* mutant larvae

(A) Heart rate measurements in *polg2*$^{+/+}$, *polg2*$^{+/ia304}$ and *polg2*$^{ia304/ia304}$ individuals at 2, 3, 4 and 8 dpf. Data are reported as heartbeats from 3 biological replicates and analysed by two-way ANOVA; *polg2*$^{+/+}$ 2 dpf (n=30), *polg2*$^{+/ia304}$ 2 dpf (n=50), *polg2*$^{ia304/ia304}$ 2 dpf (n=17); *polg2*$^{+/+}$ 3 dpf (n=30), *polg2*$^{+/ia304}$ 3 dpf (n=50), *polg2*$^{ia304/ia304}$ 3 dpf (n=16); *polg2*$^{+/+}$ 4 dpf (n=30), *polg2*$^{+/ia304}$ 4 dpf (n=50), *polg2*$^{ia304/ia304}$ 4 dpf (n=16); *polg2*$^{+/+}$ 8 dpf (n=31), *polg2*$^{+/ia304}$ 8 dpf (n=51), *polg2*$^{ia304/ia304}$ 8 dpf (n=20). (B) Light microscopy images of muscle birefringence in wt, heterozygous and homozygous *polg2*^{ia304} embryos at 3 dpf (scale bar: 400 µm). (C) Quantification of muscle birefringence in the three genotypes. Values from 3 independent biological replicates are shown as RI: Relative Intensity ± SEM and analysed by Kruskal-Wallis test followed by Dunn's multiple comparisons test; *polg2*$^{+/+}$ (n=19), *polg2*$^{+/ia304}$ (n=32), *polg2*$^{ia304/ia304}$ (n=13); * $p<0.05$.

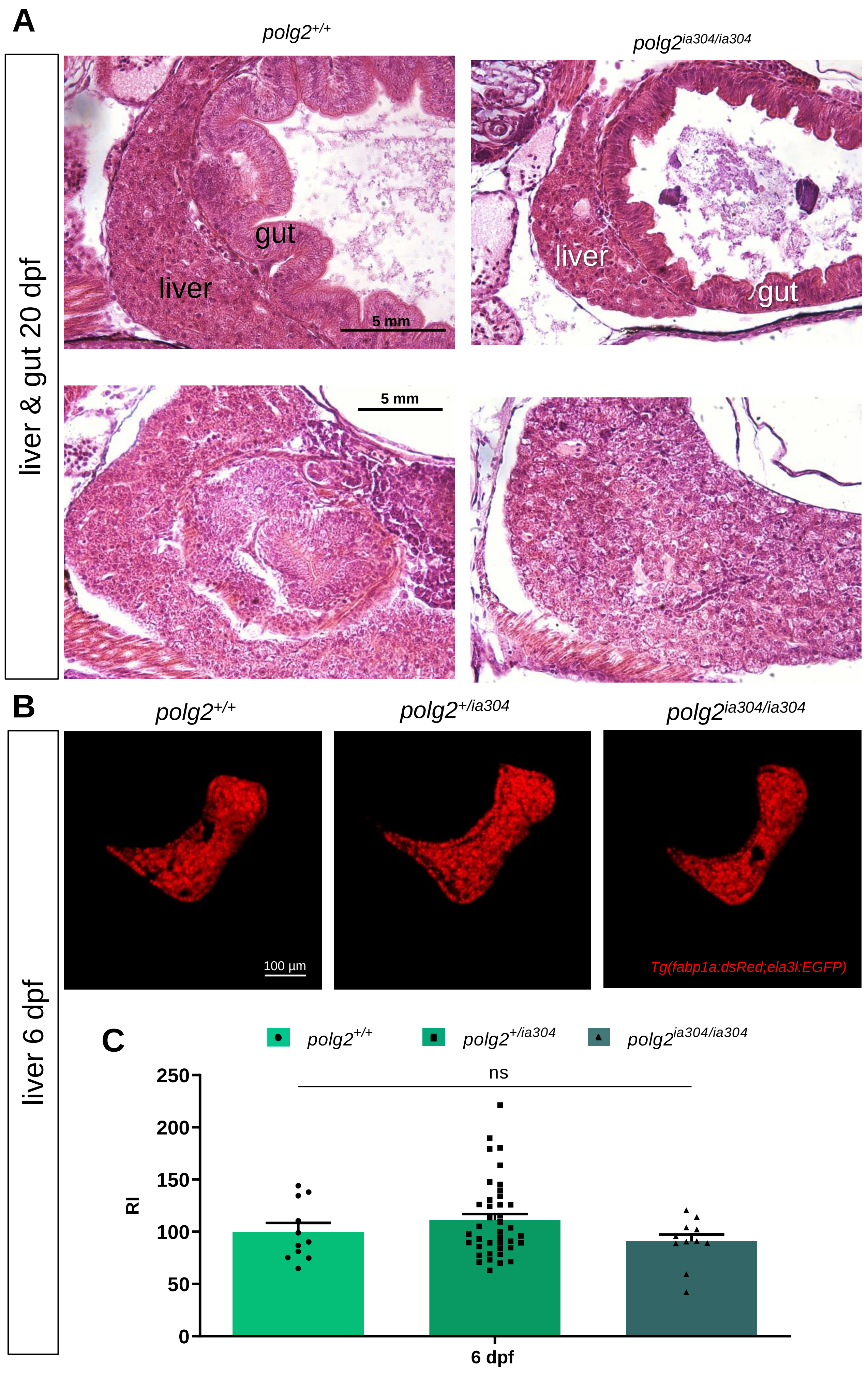

Suppl. Figure 4: Histological analysis of liver and gut in *polg2* mutants at 20 dpf.

(A) Histological analysis of gut and liver in 20 dpf zebrafish larvae. No significant alterations were found regarding their size (being isometric) and composition (scale bar: 5 mm). (B) Confocal images of the liver-expressed *Tg(lfabf:dsRed;elaA:EGFP)*^{gz15} transgene at 6 dpf (scale bar 100 µm). (C) Scatter-plot showing the relative quantification of the liver-expressed transgene in the three genotypes at 6 dpf. Data are expressed as the mean ± SEM and analysed by Kruskal-Wallis test followed by Dunn's test for multiple comparisons; *polg2*$^{+/+}$ (n=11), *polg2*$^{+/ia304}$ (n=40), *polg2*$^{ia304/ia304}$ (n=11).

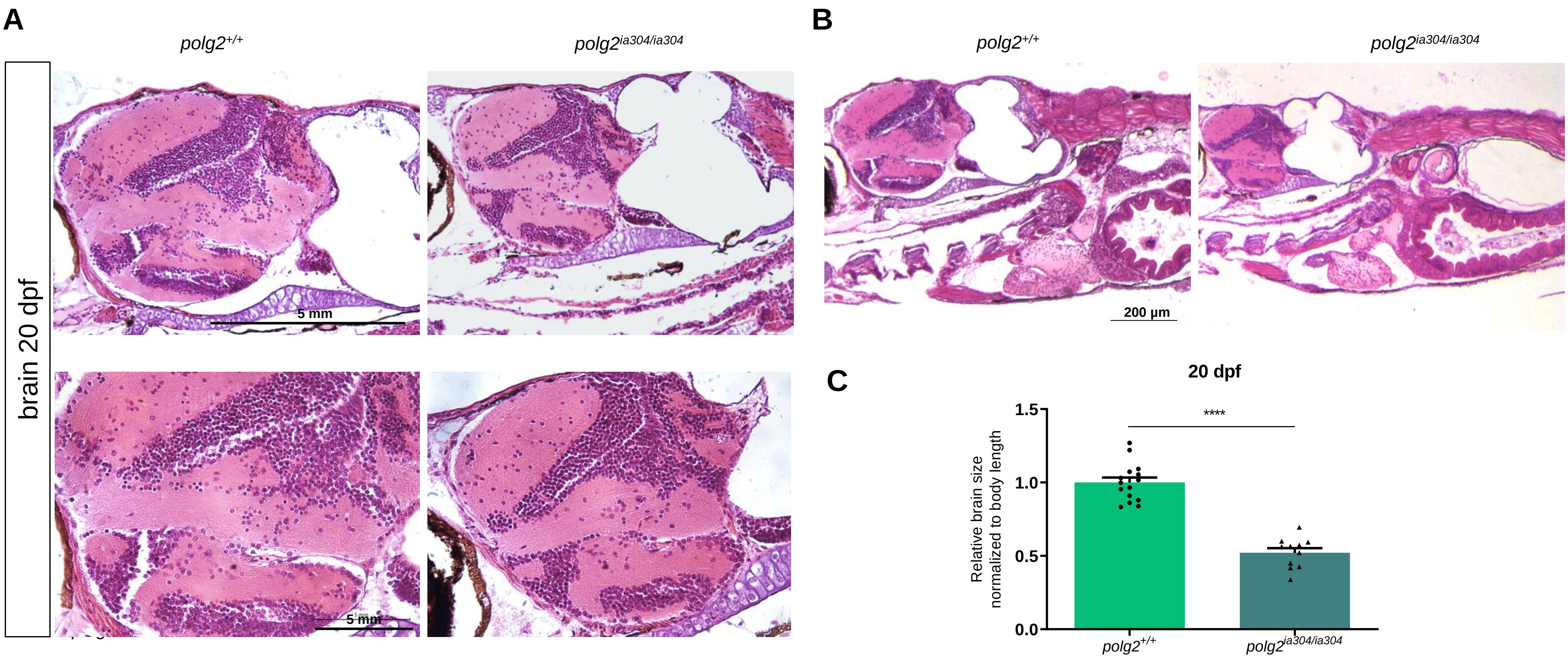

Suppl. Figure 5: Histological analysis of brain in *polg2* mutants at 20 dpf

(A) Histological sections of *polg2*$^{+/+}$ and *polg2*$^{ia304/ia304}$ brain at 20 dpf, revealing allometric reduction of the brain size in mutants (scale bar: 5 mm). (B) Histological section at lower magnification of 20 dpf *polg2*$^{+/+}$ and *polg2*$^{ia304/ia304}$ larvae (scale bar: 200 µm). (C) Scatter-plot showing the quantification of brain size normalized to body length from different sections in *polg2*$^{+/+}$ and *polg2*$^{ia304/ia304}$ at 20 dpf larvae. Values are reported as the mean ± SEM and analysed by unpaired t-test; *polg2*$^{+/+}$ (n=15), *polg2*$^{ia304/ia304}$ (n=11); **** $p<0.0001$.

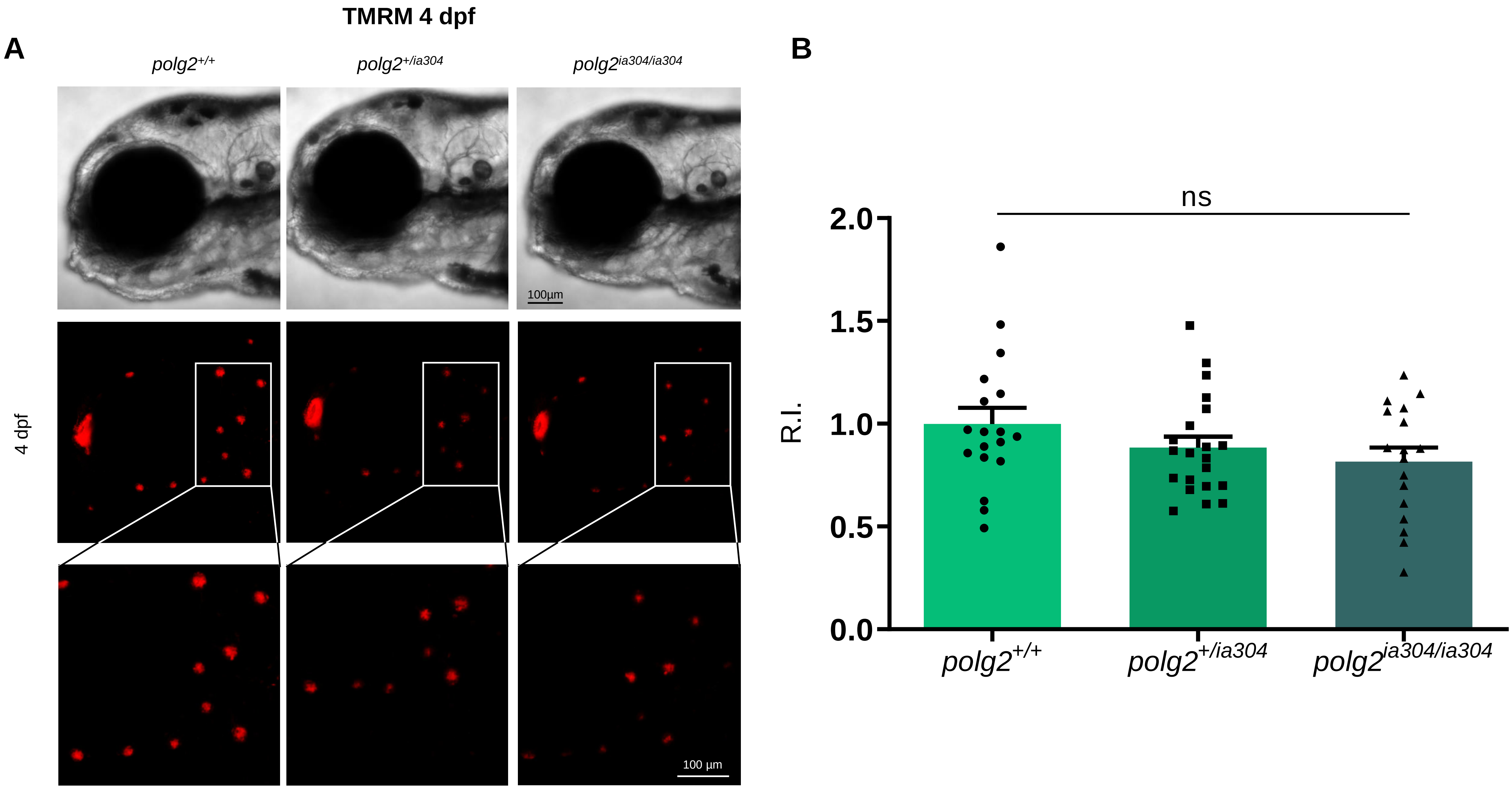

Suppl. Figure 6: Analysis of mitochondrial membrane potential in *polg2* mutant larvae.

(A) Fluorescence imaging of TetraMethylRhodamine Methyl ester (TMRM) accumulation in active mitochondria, monitored in the head of *polg2*$^{+/+}$, *polg2*$^{+/ia304}$ and *polg2*$^{ia304/ia304}$ individuals at 4 dpf (scale bar: 100 µm). (B) Quantification of mitochondrial TMRM accumulation in zebrafish embryos at 4 dpf. Data are expressed as mean ± SEM, analysed by ordinary One-way ANOVA and corrected by Tukey's multiple comparisons test; *polg2*$^{+/+}$ (n=18), *polg2*$^{+/ia304}$ (n=21), *polg2*$^{ia304/ia304}$ (n=17); RI: Relative Intensity.

Oligomer name	Gene / Accession No.	Type	Sequence (5' - 3')	Effect; Product
polg2-specific oligo	*polg* / ZDB-GENE-060303-1	CRISPR/Cas9 DNA oligomer for gRNA (**gRNA sequence**)	ATTTAGGTGACACTATA**GGGCGA TGAGAGTTTGAAAG**GTTTTAGAG CTAGAAATAGCAAG	CRISPR/Cas9-induced *polg2* mutagenesis
polg2-F	*polg2* / ZDB-GENE-060810-116	DNA primer for *ia304* genotyping	TAGGCCCGTCTGACTTCAAC	Normal product: 137 bp Deleted product: 127 bp
polg2-R	*polg2* / ZDB-GENE-060810-116	DNA primer for *ia304* genotyping	TAGTTGTGTGTCTCCCAGGG	
polg2-diagn-F	*polg2* / ZDB-GENE-060810-116	DNA primer for *ia304* sequencing	CTGACCACTGAAAGCCACTATG	Normal product: 252 bp Deleted product: 242 bp
polg2-diagn-R	*polg2* / ZDB-GENE-060810-116	DNA primer for *ia304* sequencing	ACGGATGTTTCTTGGTGAGTCT	
nucl-polg-F	*polg* / ZDB-GENE-060303-1	primer for mtDNA depletion analysis	GAGAGCGTCTATAAGGAGTAC	Reference nuclear gene Genomic DNA product: 81 bp
nucl-polg-R	*polg* / ZDB-GENE-060303-1	primer for mtDNA depletion analysis	GAGCTCATCAGAAACAGGACT	
mt-nd1-F	*mt-nd1* / ZDB-GENE-011205-7	primer for mtDNA depletion analysis	AGCCTACGCCGTACCAGTATT	Reference mitochondrial gene Mt DNA product: 143 bp
mt-nd1-R	*mt-nd1* / ZDB-GENE-011205-7	primer for mtDNA depletion analysis	GTTTCACGCCATCAGCTACTG	
mt-nd2-F	*mt-nd2* / ZDB-GENE-011205-8	primer for mtDNA depletion analysis	GCAGTAGAAGCCACCACAAA	Reference mitochondrial gene Mt DNA product: 173 bp
mt-nd2-R	*mt-nd2* / ZDB-GENE-011205-8	primer for mtDNA depletion analysis	GCTAGACCGATTTTGAGAGCC	
zf-gapdh-F	*gapdh* / ZDB-GENE-030115-1	DNA primer for Real Time RT-PCR	GTGGAGTCTACTGGTGTCTTC	Housekeeping gene cDNA control product: 161 bp
zf-gapdh-R	*gapdh* / ZDB-GENE-030115-1	DNA primer for Real Time RT-PCR	GTGCAGGAGGCATTGCTTACA	
zf-eef1a1a-F	*eef1a1a* / ZDB-GENE-030131-8278	DNA primer for Real Time RT-PCR	TGCAGAGATGGGAAAGGGT	Housekeeping gene cDNA control product: 161 bp
zf-eef1a1a-R	*eef1a1a* / ZDB-GENE-030131-8278	DNA primer for Real Time RT-PCR	GCTGGTCTCAAACTTCCACA	
polg-ex3-F	*polg* / ZDB-GENE-060303-1	DNA primer for Real Time RT-PCR	ATCTCATCCCGCTGGAAAC	Catalytic subunit gene cDNA target product: 320 bp
polg-ex5-R	*polg* / ZDB-GENE-060303-1	DNA primer for Real Time RT-PCR	GCTCATGGGAATGGGTTAAT	
polg2-ex6-F	*polg2* / ZDB-GENE-060810-116	DNA primer for Real Time RT-PCR	GCTCCATCCTGCTTTAACTCC	Accessory subunit gene cDNA target product: 141 bp
polg2-ex7-R	*polg2* / ZDB-GENE-060810-116	DNA primer for Real Time RT-PCR	GTGTCCAAGTATCCAGGCCA	

Suppl. Table 1: List of oligomers used in this study.
